# Validation in Swedish of Sydney Swallow Questionnaire

**DOI:** 10.1186/1756-0500-7-742

**Published:** 2014-10-21

**Authors:** Beatriz Arenaz Búa, Margareta Bülow

**Affiliations:** Division of Logopedics, Phoniatrics and Audiology, Jan Waldenströmsgata 18, SE- 205 02 Malmö, Sweden; Division of Ear, Nose and Throat Diseases, Head and Neck Surgery, Jan Waldenströmsgata 18, SE- 205 02 Malmö, Sweden; Department of Clinical Sciences, Lund University, Jan Waldenströmsgata 18, SE- 205 02 Malmö, Sweden; Skane University Hospital, Jan Waldenströmsgata 18, SE- 205 02 Malmö, Sweden; Diagnostic Centre of Imaging and Functional Medicine, SE- 205 02 Malmö, Sweden; Department of Clinical Sciences, Lund University, SE- 205 02 Malmö, Sweden; Skane University Hospital Malmö, SE- 205 02 Malmö, Sweden

**Keywords:** Oropharyngeal dysphagia, Validation, Questionnaire, Sydney Swallow Questionnaire

## Abstract

**Background:**

The aim of this study was to translate and adapt the Sydney Swallow Questionnaire to Swedish conditions and to evaluate the validity and test-retest reliability of the Swedish translation in patients with oropharyngeal dysphagia and in healthy controls.

**Methods:**

The validation included 20 patients with swallowing problems and 20 controls matched in age and sex. Patients were assigned a Dysphagia Outcome and Severity Scale. Content, construct, discriminant and predictive validity and test-retest reliability were evaluated.

**Results:**

The Swedish version of the Sydney Swallow Questionnaire was close to the original version, easy to fill in, and well accepted. The form fulfilled the criteria for content, construct, discriminant and predictive validity and test-retest reliability.

**Conclusions:**

The Swedish translation of the Sydney Swallow Questionnaire proved to be a valid instrument to assess dysphagia symptoms and could be used in clinical settings.

**Electronic supplementary material:**

The online version of this article (doi:10.1186/1756-0500-7-742) contains supplementary material, which is available to authorized users.

## Background

Oropharyngeal dysphagia is common in an elderly population. It might be caused by morphological changes such as tumours or inflammation, secondary to neurological diseases or the result of aging. Video-Fluoroscopic Swallow Study (VFSS), videomanometry and flexible endoscopic evaluation of swallowing (FEES) reflect changes in the physiology and biomechanics of swallowing and are valuable tools in determining the extent of dysfunction, but do not take the patient’s perspective into account.

Measurements of dysphagia severity are important when making management decisions and in the objective evaluation of treatment efficacy. Combining a self-report instrument with evaluation measures such as VFSS and FEES could contribute to these decisions.

Several questionnaires related to oropharyngeal dysphagia have been translated and validated from their original language (English) to other languages: Swallowing Quality of Life questionnaire (SWAL-QOL) [1–3] to French [4], Swedish [5], Chinese [6] and Dutch [7, 8], Eating Assessment Tool (EAT-10) [9] to Spanish [10] and Italian [11], Dysphagia Handicap Index (DHI) [12] to Portuguese [13] and Arabic [14] and MD Anderson Dysphagia Inventory (MDADI) [15] to Italian [16] and Swedish [17]. EAT-10 has been validated in patients with a wide variety of causes of dysphagia, it is simple to complete and score. DHI is a 25-item questionnaire, in which the patient can assign three responses for each question (never = 0, sometimes = 2, always = 4) resulting in a score between 0 and 100. Moreover, patients rate their dysphagia assigning a score from 0 to 7.

In Sweden there are currently two validated forms that address dysphagia symptoms: MDADI developed to assess dysphagia and quality of life in individuals with head and neck cancer and the SWAL-QOL that consists of 44 items and might be difficult for some patients to complete. We have some experience using the Self-report Symptom Inventory, known as Sydney Swallow Questionnaire (SSQ), see Additional file 1, and this is one of the reasons why we have chosen to validate it [18]. The questionnaire is well accepted, completed in a short time and less time consuming for the clinician in the everyday use, see Additional file 2. These are all important aspects to dysphagia patients and to clinicians with limited time.

When translating a form, a cross-cultural adaptation including the examination of cultural and linguistic differences is mandatory in order to obtain an equivalent instrument adapted to Swedish culture [19].

### Aim of the study

-  To translate and adapt SSQ to Swedish conditions.
-  To evaluate the validity and test-retest reliability of the Swedish translation in patients with oropharyngeal dysphagia and in healthy controls.

## Methods

### Inventory

The SSQ is a self-report inventory with a maximum total score of 1700; a visual analogue scale appears immediately beneath all but one question (Q12). Each visual analogue scale is a horizontal, 100-mm line anchored at each end by extreme statements representing normal function to the left and extreme dysfunction to the right (e.g., does not occur & occurs all the time; no difficulty & extreme difficulty). Participants were instructed to mark a single “X” across the horizontal visual analogue scale at the point which they feel best represented the severity of the particular dysfunction, thus yielding a score of 0–100 for each, corresponding to a distance in millimetres from the origin of the visual analogue scale. In addition, one investigator delivered the written instructions verbally. No attempt was made to guide the patient as to where on the visual analogue scale he/she should make the mark.

The patients answered the SSQ on two occasions: In connection with visits to the Ear Nose and Throat (ENT) clinic or to the Radiology department and at home 3 weeks later. A stamped addressed envelope was given to the subjects for the return of SSQ to the contact person of the study [18].

Those responsible for the study have received formal approval for the translation and validation from the lead author of the SSQ. The translation has been performed by back- translation. The authors separately made a first translation from English to Swedish. In phase two the items with divergent translations were discussed until a consensus was reached. In phase three the SSQ was translated back to English by an independent, native English speaker, graduated in linguistics, who did not participate in the first and second phases. In phase four the SSQ was translated back into Swedish and a pilot group of four patients with swallowing disorders and four healthy subjects completed the questionnaire. In phase five, some of the formulations in the Swedish version of the questionnaire were altered according to the comments of the pilot group [19].

### Participants

The final Swedish version was used, with approval by the ethical committee of the University of Lund (Dnr 2012/464), on 20 subjects without swallowing problems and on 20 patients with swallowing problems, both groups matched in age and sex. Information regarding the study was given to participants to obtain their written inform consent. All were older than 50 years and had adequate cognitive and language skills to comprehend study requirements. The SSQ has been validated in English in a cohort of head and neck patients [20]. None of the participants in our study had undergone previous head and neck surgery nor radiotherapy that might have influenced swallowing function. Controls were recruited when they visited the ENT department and completed the SSQ once.

Patients with oropharyngeal dysphagia for more than 3 months were included after the diagnose was confirmed with VFSS and a clinical evaluation by an otolaryngologist. After inclusion they were assigned a Dysphagia Outcome and Severity Scale (DOSS) score. This is a 7-point scale developed to systematically rate the severity of dysphagia based on VFSS and to make recommendations for diet level, independence level and type of nutrition. Level 7 is normal swallowing and level 1 stands for severe dysphagia [21].

Patients answered the SSQ twice. We included patients with neuromyogenic dysphagia and cricopharyngeal dysfunction (with and without Zenker’s diverticulum), this last group was used to measure discriminant validity.

### Statistical methods

All data were analyzed using SPSS 22 © Mac version. When a patient omitted more than 3 questions the inventory was excluded from further analysis, if 1 to 3 questions were not answered an estimated score for each omitted question was calculated based on the total score divided by the total possible score for the questions answered. Estimated scores for individual questions were only used for factor-analysis calculations, which requires a complete data set for each patient. We evaluated content, construct, discriminant and predictive validity and test-retest reliability. P values <0.05 (two-tailed) were regarded as significant [22–24].

### Content validity

Content validity and internal consistency review whether the relative importance and choice of questions within the inventory are appropriate for the intended use of the SSQ. We chose factor analysis to examine the underlying relationships between the questions and to evaluate content validity. We have used the principal-components method with the orthotran/varimax rotation. We calculated the Kaiser-Meyer-Olkin as a measure of sampling adequacy and Bartlett’s test of sphericity (if statistically significant it indicates that the relationships among the coefficients are not random). The factor analysis output presented a matrix of factor loadings. It is generally accepted that a factor loading greater than 0.3 is significant, but we selected 0.6 as cut-off for an individual question in the SSQ to be considered as part of a particular factor and it must not be represented in any other factor [18, 24]. Factor analysis provides a communality summary that gives a measure of the variance of each question that can be accounted for the combination of all the factors, which overall should account at least for 75% of the total variance of the questionnaire. The total variance that each question contributes should be more than 0.6 [18, 24].

### Construct, discriminant and predictive validity

Construct validity refers to whether an instrument measures the true clinical state of the patient. We hypothesized that the DOSS correlated with the SSQ and used Spearman’s nonparametric correlations to confirm this.

Discriminant validity measures the SSQ ability to distinguish clinically significant differences in therapeutic responses over time, e.g. pre and postoperative scores.

We compared the SSQ score, using the Wilcoxon test, pre-operatively and 4 week post-operatively in 4 patients with Zenker’s diverticulum treated with staples myotomy and 6 with cricopharyngeal dysfunction treated with balloon dilatation.

Predictive validity or known-groups validity refers in this case to whether SSQ can differentiate between patients with dysphagia and normal swallowers or patients with different severity of dysphagia. We have used the Mann Whitney U test to evaluate predictive validity.

### Test-retest reliability

The test–retest reliability measures the ability of the SSQ to yield consistent scores over time, given that the clinical status of the patient remains stable. We evaluated the variability of the score within 3 weeks time using the Intraclass Correlation Coefficient (ICC) and limits of agreement (LOA), which is the 95% confidence intervals of the mean of the individual differences between test and retest [22].

Ceiling and floor effects were assessed. A ceiling effect is said to occur when a high proportion of subjects in a study have maximum scores on the observed variable (the opposite is called floor effect). This makes discrimination among subjects on the top or the lower end of the scale impossible [25].

## Results

### Descriptive statistics

We recruited 20 controls and 20 patients with dysphagia, 10 men and 10 women, mean age 72 years in both groups. All responded the SSQ in less than 10 min. One patient did not answer one question and one patient did not submit the postoperative questionnaire. None of the participants experienced difficulties in completing the questionnaire.

### Content validity (internal consistency)

Kaiser-Meyer-Elkin was 0.75 indicating a sufficient sample size for the number of questions in the questionnaire. Bartlett’s test of sphericity was significant p <0.001.

The factor analysis matrix, showed that all questions except Q12 contributed significantly to factor 1 (Table 1). Question 12 (related to how long time does it take to eat) was the sole contributor to factor 3. All questions had a communality loading >0.6 and 85% of the variance in response is explained by the 4 major factors identified by the analysis, 61% for the first factor (dysphagia).

**Table 1:** **Summary of the factor analysis matrix with communality summary in patients, n = 20**

Question	Factor 1 dysphagia	Factor 2	Factor 3	Factor 4	Communality summary loading
1	0.788	0.388	0.239	-0.106	0.840
2	0.837	-0.345	-0.184	-0.153	0.877
3	0.809	-0.362	0.066	-0.312	0.887
4	0.889	-0.248	0.189	-0.079	0.893
5	0.805	0.176	0.424	-0.249	0.922
6	0.786	0.343	0.196	-0.282	0.852
7	0.622	0.455	-0.498	0.170	0.871
8	0.625	0.498	0.120	0.418	0.827
9	0.765	0.124	-0.003	-0.258	0.667
10	0.781	0.099	0.000	0.449	0.821
11	0.858	-0.293	-0.225	0.243	0.932
12	0.336	-0.287	0.759	0.346	0.891
13	0.812	-0.452	-0.036	0.251	0.928
14	0.853	0.030	-0.227	0.067	0.785
15	0.822	-0.322	-0.310	-0.012	0.876
16	0.914	0.175	0.039	0.050	0.871
17	0.804	0.140	-0.166	-0.230	0.747
Variance	61.2	9.5	8.3	6.2	

### Construct, discriminant and predictive validity

**Figure 1:**
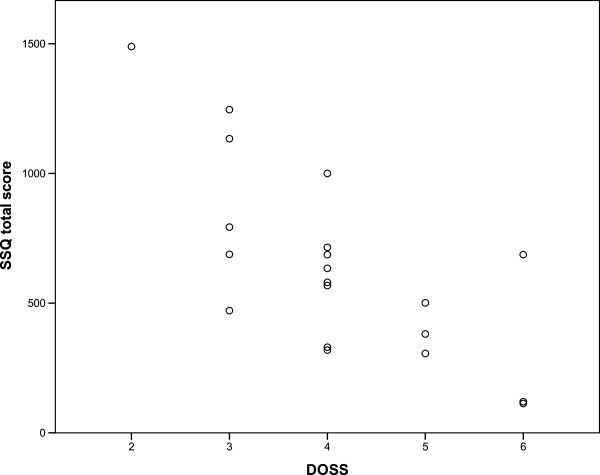
**Construct validity; Spearman’s correlation between DOSS and the SSQ total score.**

**Figure 2:**
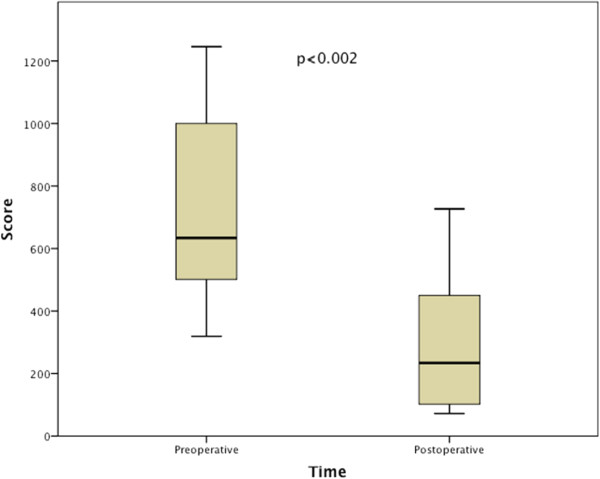
**Discriminant validity; Wilcoxon signed rank test, showing pre-operative and 1 month post-operative comparison.**

**Figure 3:**
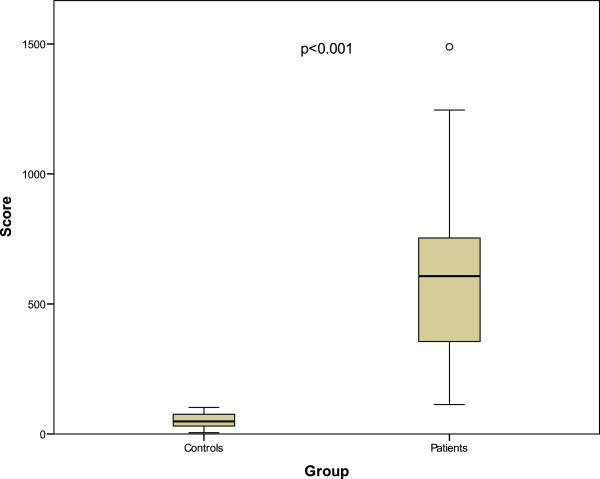
**Predictive validity; Mann–Whitney U test, comparison between participants with and without dysphagia.**

Spearman correlations coefficient was -0.70, p < 0.001 confirming construct validity (Figure 1).Regarding discriminant validity the preoperative mean value was 722, median 634 and postoperative mean 313, median 234 and the Wilcoxon signed ranks test was significant with p =0.002 (Figure 2).Predictive validity: as hypothesized, dysphagic patients scored significantly higher on SSQ, p < 0.001. The mean score for controls was 51, median 48; minimum score was 5 and maximal 102. The mean score for patients was 638, median 607; minimum score was 113 and maximal 1489 (Figure 3).

### Test-retest reliability

The ICC for patient scores within 3 weeks was 0.98, 95% CI (0.96-0.99) significant p < 0.001 (Table 2), 5 questions had ICC <0.7: Q1 0.63, Q3 0.64, Q8 0.53 and Q12 0.61.

**Table 2:** **Summary of the test–retest reliability, using the Intraclass Correlation Coefficient (ICC), confidence interval (CI)**

	ICC	95% CI	P-value
Total score	0.98	0.95-0.99	< 0.001
Q1	0.63	0.27-0.83	0.001
Q2	0.86	0.28-0.84	< 0.001
Q3	0.64	0.29-0.84	0.001
Q4	0.94	0.85-0.97	<0.001
Q5	0.88	0.72-0.95	<0.001
Q6	0.93	0.82-0.97	<0.001
Q7	0.85	0.65-0.94	<0.001
Q8	0.53	0.12-0.78	0.007
Q9	0.74	0.45-0.89	<0.001
Q10	0.87	0.71-0.95	<0.001
Q11	0.76	0.50-0.89	<0.001
Q12	0.62	0.25-0.83	0.002
Q13	0.91	0.79-0.96	<0.001
Q14	0.80	0.57-0.92	<0.001
Q15	0.93	0.83-0.97	<0.001
Q16	0.75	0.47-0.89	<0.001
Q17	0.81	0.58-0.92	0.001

## Discussion

A self-report instrument is commonly used to assess patient reported outcome, it guarantees that questions are asked in a standardized manner, and facilitates comparisons within and between groups. These inventories are designed to measure either health-related quality of life (HRQoL) or functional health status (FHS), HRQoL refers to the perception individuals may have on their health taking into account social, functional and psychological issues, whereas FHS quantifies the symptomatic severity of a disease (in this case dysphagia) on particular functional aspects. DHI, MDADI and SWAL-QOL are HRQoL questionnaires, EAT-10 and SSQ are FHS questionnaires. For optimal use of both types of inventories, it is necessary to combine psychometric and utility approaches [26–28].

The Swedish version of SSQ was well accepted, the response rate was high, and the number of missing items was very low (only Q6 in patient 19). The results indicated that the translation of SSQ was easy to manage and close to the original. It took less time to answer (less than 10 minutes) and score (less than 4 minutes), compared to SWAL-QOL and the MDADI. It had good test-retest reliability. The ICC for the total score was 0.98. All the questions reached the level 0.7 except Q1 (grade of dysphagia), Q3 (difficulty to swallow thick liquids), Q8 (difficulty to initiate the swallowing) and Q12 (how long does it take to eat), (Table 2).

Our sample was small which might be a limitation in our study, but the Kaiser-Meyer-Olkin was 0.75, indicating a sufficient sample for the number of questions in our questionnaire. The Swedish SSQ satisfied as well criteria for content, construct, discriminant, and predictive validity. Regarding content validity four factors accounted for 85% of its variance, the dominant factor (dysphagia) accounted for 61%, slightly better than in the original article that was 59%.

The total inventory score showed a -0.70 correlation with the DOSS, showing excellent construct validity. The correlation is negative: DOSS score decreases (level 1) and SSQ score increases (maximum 1700) when dysphagia severity rises. Wallace et al. calculated the construct validity correlated the SSQ with a global assessment score obtaining a positive linear correlation 0.69 [18].

Discriminant validity is important for using the inventory to measure responses to treatment and to establish the efficacy of that treatment. The mean preoperative total score decreased by an average of 60% postoperatively, 10% less than in the original article, but our study includes patients treated both with myotomy and balloon dilations and they probably differ in their postoperative results.

Subjects with dysphagia had significantly higher scores than the age- and gender-matched control group, suggesting very good predictive validity that helps to distinguish between individuals with/without dysphagia, which is central in a broader use. The cut-off score for dysphagia in our version of SSQ is 111, this is the controls mean total score plus two standard deviations (51 + (2 × 30) ≥ 111). Score values higher than 111 should be considered as pathological in our validation. However, in Wallace et al. SSQ the cut-off score is 193 for 19 controls with a mean age of 62.

Floor and ceiling effects were not found in the Swedish SSQ. They were not reported in the SSQ original version.

By performing a Swedish version and validation of SSQ we have obtained a useful tool to record patient reported outcome of swallowing problems. This self-report instrument is not only easy for the patients to use, but also very efficient for the clinician.

## Conclusions

The Swedish version of the SSQ seems to be a reliable and consistent instrument for the assessment of subjective dysphagia symptoms. The availability of validated patient reported outcome instruments such as the SSQ might be an important contribution to both research and screening of dysphagia in Sweden.

## Electronic supplementary material

Additional file 1: **Original Sydney Swallow Questionnaire.** (DOC 30 KB)

Additional file 2: **Swedish version of the Sydney Swallow Questionnaire.** (DOCX 100 KB)

## Abbreviations

CI: Confidence intervals
DHI: Dysphagia Handicap Index
DOSS: Dysphagia Outcome and Severity Scale
EAT-10: Eating Assessment Tool
ENT: Ear Nose and Throat
FEES: Flexible Endoscopic Evaluation of Swallowing
FHS: Functional Health Status
HRQoL: Health-Related Quality of Life
ICC: Intraclass Correlation Coefficient
MDADI: MD Anderson Dysphagia Inventory
SWAL-QOL: Swallowing Quality of Life Questionnaire
SSQ: Sydney Swallow Questionnaire
VFSS: Video-Fluoroscopic Swallow Study
Q: Question.
