# Supplementary material for: Validation in Swedish of Sydney Swallow Questionnaire
Source: BMC Res Notes. 2014 Oct 21;7:742. doi: 10.1186/1756-0500-7-742 (PMC4216845; doi:10.1186/1756-0500-7-742)
Supplement: Supplementary file 1 — Additional file 1: Original Sydney Swallow Questionnaire. (DOC 30 KB) [file 13104_2014_3276_MOESM1_ESM.doc]

**SYDNEY SWALLOW QUESTIONNAIRE**

1. How much difficulty do you have swallowing at **present**?
2. How much difficulty do you have **swallowing THIN liquids** do you have? (e.g. tea, juice, beer, coffee)
3. How much difficulty do you have **swallowing THICK liquids**? (e.g. milkshakes, soups, custard)
4. How much difficulty do you have **swallowing SOFT foods**? (e.g. mornays, scrambled egg, mashed potato)
5. How much difficulty do you have **swallowing HARD foods**? (e.g. steak, raw fruit, raw vegetables)
6. How much difficulty do you have **swallowing DRY foods** (e.g. bread, biscuits, nuts)
7. Do you have any difficulties to **swallowing your own saliva**?
8. Do you ever have difficulty **starting a swallow**?
9. Do you ever have a **feeling of food** getting **stuck** in the throat when you swallow?
10. Do you ever **cough or choke** when swallowing **solid foods**? (e.g. bread, meat or fruit)
11. Do you ever **cough or choke** when swallowing **liquids**? (e.g. coffee, tea, water, beer)
12. How long does it take to **eat an average meal**?
13. When you swallow does food or liquid ever go **up behind your nose or come out of your nose?**
14. Do you ever need to **swallow more than once** for food go down?
15. Do you ever **cough up or spit out food or liquids DURING a meal**?
16. How do rate the **severity of your swallowing problem today**?
17. How much does your swallowing problem **interfere with your enjoyment or quality of life**?
