# Supplementary material for: Validation in Swedish of Sydney Swallow Questionnaire
Source: BMC Res Notes. 2014 Oct 21;7:742. doi: 10.1186/1756-0500-7-742 (PMC4216845; doi:10.1186/1756-0500-7-742)
Supplement: Supplementary file 2 — Additional file 2: Swedish version of the Sydney Swallow Questionnaire. (DOCX 100 KB) [file 13104_2014_3276_MOESM2_ESM.docx]

**Sydney Swallow Questionnaire (Swedish version)**

1. Hur stora svårigheter har Du för NÄRVARANDE att kunna svälja?

2. Hur stora svårigheter har Du att kunna svälja TUNNFLYTANDE vätskor?(t ex vatten, te, saft, kaffe)

3. Hur stora svårigheter har Du att kunna svälja TRÖGFLYTANDE vätskor? (t ex fruktsoppor, filmjölk, yoghurt, vaniljsås)

4. Hur stora svårigheter har du att svälja MJUK, SLÄT KOST?

(t ex potatismos, äggröra, gröt, purémat)

5. Hur stora svårigheter har du att svälja FAST föda (normal kost)?(t ex kött, frukt, grönsaker, ris)

6. Hur stora svårigheter har du att svälja TORR föda?

(t ex bröd, kakor, nötter)

7. Har du några svårigheter att kunna SVÄLJA DIN SALIV?

8. Har du några svårigheter att KOMMA IGÅNG OCH SVÄLJA

(påbörja en sväljning)

9. Har du någon gång EN KÄNSLA AV ATT MAT HAKAR UPP SIG

(fastnar) i halsen när du sväljer?

10. HOSTAR DU ELLER SÄTTER I HALSEN när du sväljer fast föda?(t ex bröd, kött eller frukt)

11. HOSTAR DU ELLER SÄTTER I HALSEN när du sväljer VÄTSKOR? (t ex kaffe, te, vatten, öl)

12. Hur lång tid tar det för dig ATT ÄTA EN VANLIG MÅLTID?

13. Händer det att mat eller VÄTSKA KOMMER UPP I NÄSAN eller KOMMER UT UR NÄSAN när du sväljer?

14. Behöver du någon gång SVÄLJA MER ÄN EN GÅNG för att födan skall kunna sväljas ner?

15. Händer det någon gång att du HOSTAR UPP ELLER

SPOTTAR UT MAT ELLER VÄTSKA UNDER EN MÅLTID? 16. Hur ALLVARLIGA bedömer du att dina SVÄLJNINGSPROBLEM ÄR IDAG?

17. Hur MYCKET påverkar dina sväljningsproblem DIN LIVSGLÄDJE OCH DIN LIVSKVALITÉ?
